# MUTACLASH: identifying functional small RNA target sites using crosslinking-induced mutations

**DOI:** 10.1261/rna.080482.125

**Published:** 2026-02

**Authors:** Wei-Sheng Wu, Dong-En Lee, Chi-Jung Chung, Shang-Yi Lu, Jordan S. Brown, Donglei Zhang, Heng-Chi Lee

**Affiliations:** 1 Department of Electrical Engineering, National Cheng Kung University, Tainan 701, Taiwan; 2 Department of Molecular Genetics and Cell Biology, University of Chicago, Chicago, Illinois 60637, USA

**Keywords:** Argonaute, CLASH, PIWI, piRNA, small RNA

## Abstract

Small RNAs play essential roles in gene regulation across diverse biological processes. Crosslinking, ligation, and sequencing of hybrids (CLASH) experiments have revealed that PIWI and Argonaute proteins can each bind a wide range of mRNA targets with distinct base-pairing rules, raising questions about the flexibility and functional relevance of these interactions. Given that crosslinking-induced mutations (CIMs) provide single-nucleotide resolution molecular footprints of RNA-binding proteins, we developed MUTACLASH, a bioinformatics tool for systematically analyzing CIMs in CLASH data sets. Our analyses indicate that CIMs function as molecular footprints of Argonaute binding on target mRNAs. Specifically, for *Caenorhabditis elegans* miRNA and piRNA CLASH data, CIMs are enriched at the center of small RNA binding sites, as well as at nucleotides within mRNA target sites that exhibit local mismatches in piRNA interactions. Furthermore, we show that mRNAs with noncanonical miRNA and piRNA binding sites and/or low hybrid abundance marked by CIMs exhibit stronger regulatory effects than those without CIMs, demonstrating the utility of CIM analysis in identifying functional small RNA binding sites, including those that are otherwise likely overlooked with current analysis tools.

## INTRODUCTION

Distinct types of small RNAs, such as miRNAs and piRNAs, are critical regulators of gene expression in diverse animals (Höck and Meister 2008). miRNAs and piRNAs are essential regulators of gene expression, with miRNAs primarily guiding Argonaute proteins to posttranscriptionally repress mRNAs in diverse cellular processes, while piRNAs, in complex with PIWI Argonaute family proteins, play a crucial role in maintaining genome integrity by silencing transposable elements and regulating gene expression in germ cells (Ambros 2004; Siomi et al. 2011). Both types of small RNAs guide Argonaute family proteins to recognize mRNA targets through base-pairing interactions between small RNAs and mRNAs (Höck and Meister 2008; Hutvagner and Simard 2008). For miRNA, pairing between the seed region, the second to the seventh or eighth nucleotide of small RNAs, and the target mRNA is reported to play a critical role in target recognition of animal miRNA and piRNAs (Shin et al. 2010; Zhang et al. 2018).

While reported piRNA targeting rules in *Caenorhabditis elegans* do not tolerate seed mismatches (Zhang et al. 2018), few recent studies have suggested that piRNAs can tolerate a seed mismatch in target recognition to trigger gene silencing in *C. elegans* and in other animals (Priyadarshini et al. 2022; Gainetdinov et al. 2023; Ariura et al. 2024; Li et al. 2024). Since both canonical (perfect seed pairing) and noncanonical (imperfect seed pairing) interactions can lead to gene silencing by miRNA and piRNA, predicting functional small RNA target sites remains challenging (Brennecke et al. 2005; Gainetdinov et al. 2023). These observations suggest that base-pairing rules alone cannot fully explain target recognition by PIWI or Argonaute proteins, raising intriguing questions about the molecular mechanisms that enable these proteins to distinguish and adapt to distinct modes of target binding.

Crosslinking, ligation, and analysis of sequence hybrid (CLASH) is a critical experimental approach for the identification of small RNA binding sites in vivo (Helwak et al. 2013). In CLASH experiments, UV crosslinking leads to the formation of covalent bonds between Argonautes and their interacting mRNAs, allowing for a stringent purification process. The small RNAs are then ligated to partially degraded target mRNAs, which are then cloned into cDNA libraries and sequenced. Analysis of hybrid reads consisting of a small RNA and its target RNA can, therefore, reveal the transcriptome-wide interactions between small RNAs and their target mRNAs (Helwak et al. 2013). Surprisingly, the majority of hybrid reads from CLASH data do not contain canonical small RNA–mRNA interaction (Helwak et al. 2013; Broughton et al. 2016; Shen et al. 2018). Further analyses of miRNA binding sites with noncanonical interactions suggest that most of these interactions have limited functional relevance (Agarwal et al. 2000). Nonetheless, empirical studies of small RNA-mediated gene regulation have demonstrated that noncanonical small RNA binding sites can be of functional significance (Vella et al. 2004; Brennecke et al. 2005; Brancati and Großhans 2018). Therefore, developing strategies to identify functional candidates among noncanonical binding sites in CLASH data is crucial, as distinguishing biologically relevant interactions from background noise remains a significant challenge.

Crosslinking-induced mutations (CIMs) arise during cDNA synthesis in UV crosslinking of crosslinking and immunoprecipitation (CLIP) experiments due to the covalent attachment of RNA-binding proteins (RBPs) to their target RNA upon UV irradiation. This crosslinking event creates a physical barrier that disrupts the normal progression of reverse transcriptase (RT) during cDNA synthesis, leading to characteristic CIMs, including deletion or substitutions at/around the crosslinked site (Zhang and Darnell 2011). Given that CIM formation requires direct and immediate proximity between the mRNA and RNPs, CIMs have been shown to act as the molecular footprint of RNA–protein interactions at single-nucleotide resolution (Zhang and Darnell 2011). Therefore, analyzing CIMs distribution offers valuable insights into the binding conformations and target recognition mechanisms of PIWI and Argonaute proteins of small RNA pathways. While currently there are several tools to analyze CLASH data (Travis et al. 2014; Zhong and Zhang 2019; Videm et al. 2021; Wu et al. 2022), they do not consider CIMs. Here, we developed the MUTACLASH analysis pipeline to identify CIMs and examined CIM distribution from piRNA and miRNA CLASH data in *C. elegans*. Our results show that CIMs are enriched at the center of small RNA binding sites and at mismatched nucleotides within piRNA target sites. Importantly, piRNA and miRNA binding sites with CIMs generally exhibit stronger regulatory effects than those without CIMs, including those without canonical base-pairing interactions and/or those target sites of low hybrid abundance. This suggests that CIMs mark functionally relevant small RNA binding events, providing an additional metric other than pairing score/energy or hybrid abundance for distinguishing regulatory interactions from background binding in CLASH data. Together, our CIM analysis of CLASH data maps the crosslinking landscape of piRNA–PIWI and miRNA–Argonaute interactions, offering insights into PIWI and Argonaute target recognition. Furthermore, our results demonstrate that CIM analysis with MUTACLASH provides comprehensive insights into assessing the functional significance of CLASH-identified small RNA binding sites, providing candidates that are likely being overlooked through current analysis tools.

## RESULTS

### Identification of CIMs from PIWI and Argonaute CLASH data

**FIGURE 1.**
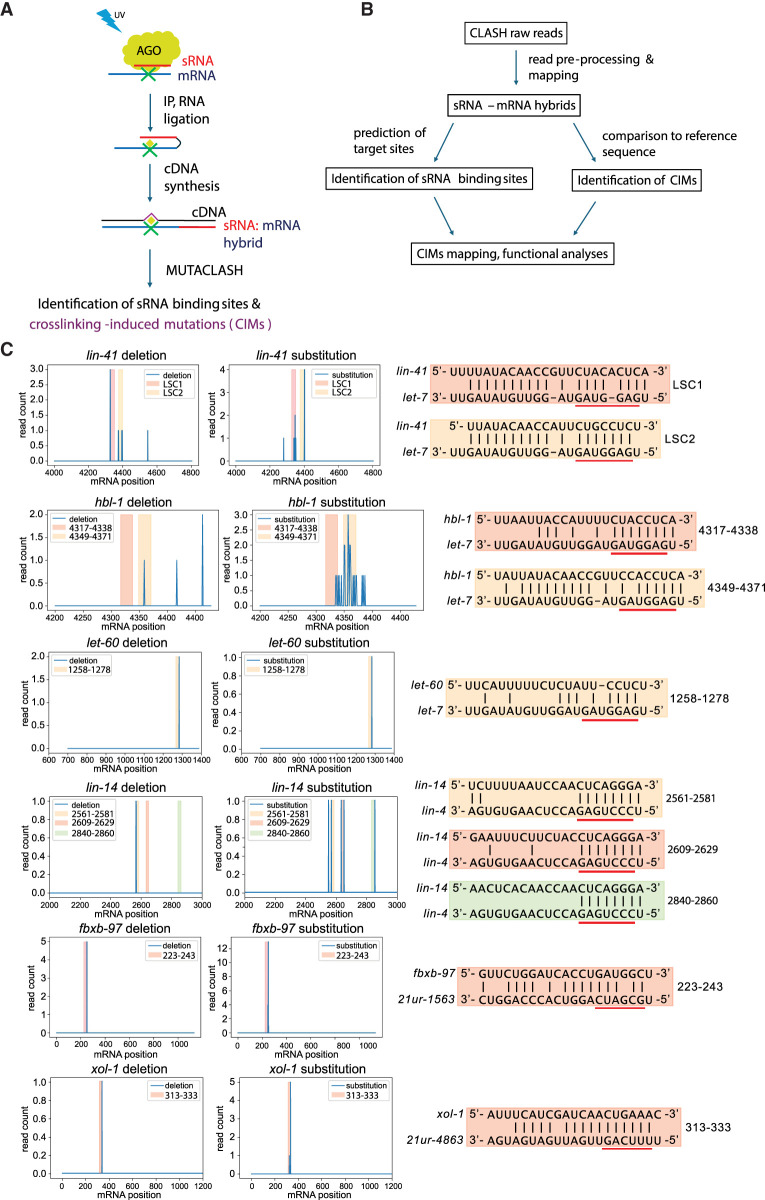
Identification of crosslinking-induced mutations (CIMs) in CLASH data at piRNA and miRNA targeting sites. (*A*) A model illustrating the CLASH experimental procedure of PIWI and Argonaute complexes and the production of CIMs during cDNA synthesis. (*B*) A flowchart of MUTACLASH analysis pipeline for identifying small RNA–mRNA hybrids and for analyzing CIMs from PIWI and Argonaute CLASH data. (*C*) The distribution of deletions (*left*) and substitutions (*middle*) on targeting mRNAs identified from hybrid reads from ALG-1 Argonaute iCLIP data (*let-7* and *lin-14*) or PRG-1 PIWI CLASH data (*21ur-1563* and *21ur-4863*). The base-pairing between miRNA (*let-7* and *lin-14*) or piRNA (*21ur-1563* and *21ur-4863*) and the targeting mRNAs is shown (*right*). The seed region of miRNA (*let-7* and *lin-14*) or piRNA (*21ur-1563* and *21ur-4863*) is underlined.

As mentioned above, CIMs represent the footprints of RNA binding proteins on their target mRNA in CLIP experiments (Zhang and Darnell 2011). Since both CLIP and CLASH experiments involve UV crosslinking, we reasoned that CIMs should also be present in PIWI/Argonaute CLASH data (Fig. 1A) and those crosslinking sites likely represent the footprint of PIWI/Argonaute on their target mRNAs.

To examine whether the CIMs, such as mRNA deletion or substitution, are present in CLASH data, we developed the MUTACLASH analysis pipeline that takes raw sequencing reads from CLASH data that integrate those previously published algorithms to identify small RNA–mRNA hybrids (Travis et al. 2014; Zhong and Zhang 2019; Videm et al. 2021; Wu et al. 2022) and critically, to locate CIMs within these hybrids (Fig. 1B). To ensure that CIMs are not mistakenly reported due to alignment ambiguity—particularly for mRNAs with highly similar sequences, such as those derived from transposon variants—MUTACLASH incorporates a filtering step that removes CIM annotations when the corresponding mRNA sequence can perfectly align to alternative genomic or transposon-derived sequences (Supplemental Fig. S1A–C). By analyzing PIWI PRG-1 (piRNA) CLASH and Argonaute ALG-1 (miRNA) iCLIP (a CLASH-like method) data sets in *C. elegans* (Broughton et al. 2016; Shen et al. 2018), we first examined several previously well-characterized piRNA and miRNA targeting sites. These include the following: miRNA *let-7* binding sites on *lin-41*, *hbl-1*, and *let-60* mRNA targeting sites, miRNA *lin-4* binding sites on *lin-14* mRNA*,* piRNA *21ur-1563* binding site on mRNA *fbxb-97*, and piRNA *21ur-X1* or *21ur-4863* binding sites on mRNA *xol-1* (Vella et al. 2004; Johnson et al. 2005; Broughton et al. 2016; Shen et al. 2018; Ilbay and Ambros 2019). We found CIMs including deletions and/or substitutions, in hybrid reads at these targeting sites (Fig. 1C), except for hybrids formed between 21ur-X1 and xol-1 mRNA, which did not contain CIMs. Notably, CIMs are present at both the canonical target sites with perfect seed pairing, such as the *let-7* miRNA target site LSC2 on *lin-41* miRNA, as well as the noncanonical target sites with seed mismatch or bulge, such as the *let-7* miRNA target site LSC1 on *lin-41* mRNA or the *21ur-1563* piRNA target site on *fbxb-97* mRNA (Fig. 1C). These observations supported that CIMs are frequently present at piRNA PIWI and miRNA Argonaute CLASH data.

To assess whether these mutations identified from MUTACLASH are indeed induced by crosslinking, we first compared the mutations of mRNAs from CLASH reads with those from mRNA sequencing reads, which are obtained with and without UV crosslinking in their experimental procedures, respectively. Our analysis indicated that mRNA derived from PRG-1 and ALG-1 CLASH data exhibited a higher incidence of deletions (6.65% and 5.8%) or substitutions (6.76% and 7.41%) compared to deletions (0.026%) and substitutions (1.4%) from mRNA sequencing data (Table 1). While the percentage of mRNA segments in hybrid reads containing CIMs is slightly lower than that in mRNA (non-hybrid) reads from PRG-1 CLASH data (2.8% deletion and 3.27% substitution), it remains significantly higher than what is observed in mRNA sequencing data (Table 1).

**TABLE 1.** Frequency of reads with mRNA deletion or substitution in RNA sequencing data or CLASH data

Library	Read type	# Reads with deletion	# Reads with substitution	# Total reads	% Reads with mutation
mRNA	Non-hybrid (mRNA only)	5166.5	281,215	20,038,571	Deletion: 0.026% Substitution: 1.40%
PRG-1 CLASH	Non-hybrid (mRNA only)	265,382	270,426.5	4,053,251	Deletion: 6.65% Substitution: 6.76%
ALG-1 iCLIP	Non-hybrid (mRNA only)	875,559.5	1,120,123	15,107,177	Deletion: 5.8% Substitution: 7.41%
PRG-1 CLASH	Hybrid	108,970	129,279	4,017,410	Deletion: 2.8% Substitution: 3.27%
ALG-1 iCLIP	Hybrid	4897	6155	133,974	Deletion: 4.59% Substitution: 3.67%

Because highly abundant hybrids might be more likely to contain CIMs, we next asked whether CIM occurrence was merely a consequence of hybrid abundance. If this were the case, we would expect a strong correlation between the number of CIM-containing hybrids and the total number of hybrids from each small RNA target site. To examine this, we aggregated read counts for each small RNA targeting site (see Materials and Methods for more details) and compared CIM-containing hybrid abundance with total hybrid abundance. Our analyses revealed only moderate correlations (Supplemental Fig. S2A–D, *R* = 0.245–0.403). Furthermore, in our analysis of the regulatory effects of small RNA target sites (see later in the manuscript), we found that the presence of CIMs provided a valuable addition for assessing regulatory impact. Together, our observations suggest that although there is a positive relationship between hybrid abundance and the CIM presence, CIM presence is not solely determined by hybrid abundance.

In addition, we observed that CIMs identified from PRG-1 or ALG-1 CLASH data, both non-hybrid and hybrid reads, exhibited a preference for uridine-derived mutations (ranging from 42% to 64%) (Supplemental Table S1A). On the contrary, no such uridine preference was found in mRNA sequencing data. These findings align with previous reports that uridine has higher photo-reactivity and thus is preferentially crosslinked to proteins during UV crosslinking (Sugimoto et al. 2012; Zaman et al. 2015). Moreover, we observed a strong preference for specific types of substitutions in PRG-1 and in ALG-1 CLASH data, including U to C substitutions (representing 83%–90% of all U substitutions) and C to U substitutions (representing 84% of all C substitutions) (Supplemental Table S1B,C). Collectively, the elevated mutation frequency and mutation preference confirmed that CIMs are present in CLASH data.

### CIMs are enriched at nucleotides corresponding to the center of piRNA and miRNA binding sites

**FIGURE 2.**
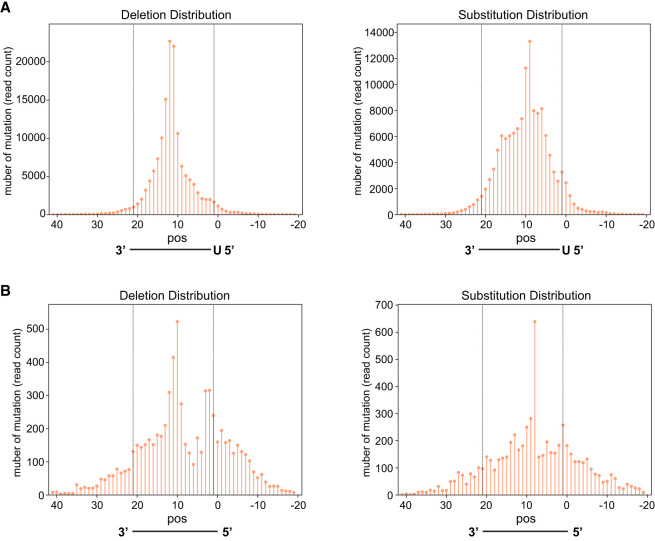
The distribution of CIMs at piRNA and miRNA targeting sites. (*A*) The number of deletions (*left*) and substitutions (*right*) found at the indicated position (pos) of mRNAs corresponding to their targeting piRNAs from PRG-1 piRNA CLASH data. (*B*) The number of deletions (*left*) and substitutions (*right*) found at the indicated position (pos) of mRNAs corresponding to their targeting miRNA ALG-1 miRNA Argonaute iCLIP data.

Previous analyses of CIMs for CLIP experiments of various RNA-binding proteins have shown that CIMs are preferentially located at specific positions within their mRNA binding motifs (Feng et al. 2019). Unlike sequence-specific RNA-binding proteins, PIWI and Argonaute family proteins are guided by small RNAs of distinct sequences to target mRNAs. CLIP analysis of neuronal tissue has implied that CIMs may occur in the middle of the miRNA pairing site (Zhang and Darnell 2011). However, since CLIP data identify mRNA targets but do not reveal their corresponding miRNAs, the pairing information and CIM locations can only be inferred rather than directly determined. Therefore, it remains unclear whether CIMs are enriched at specific positions of PIWI and Argonaute binding sites. CLASH data offer the ideal data set to examine the distribution of CIMs in Argonaute binding sites as hybrid reads provide both the identity of mRNAs and targeting miRNAs (Helwak et al. 2013). We applied MUTACLASH to analyze PRG-1 piRNA CLASH data and to calculate the distribution of CIMs at single-nucleotide resolution across the entire piRNA targeting sites (Shen et al. 2018). We found that CIMs detected in mRNAs were located preferentially at nucleotides corresponding to the center of piRNA–mRNA interactions (Fig. 2A). Specifically, mRNA deletions were peaked at positions corresponding to the 11th and 12th nucleotides of piRNAs, while the mRNA substitutions were peaked at positions corresponding to the ninth and 10th nucleotides of piRNAs (Fig. 2A). This enrichment of CIMs at the center region was even more pronounced when we only consider those piRNA target sites within the top 33% piRNA targeting scores (Supplemental Fig. S3A; Wu et al. 2018). Of those sites with high piRNA targeting scores, a minor peak of CIMs can also be found at the beginning of piRNAs.

We then analyzed the position of CIMs in miRNA (ALG-1 Argonaute) CLASH data of *C. elegans* (Broughton et al. 2016). Interestingly, several of the trends of CIM distribution observed in piRNA were also found in miRNAs. First, CIMs were also enriched at nucleotides corresponding to the center and slightly at the beginning of their target sites (Fig. 2B). The peak positions of deletions and substitutions are also enriched at the 11/12th and 9/10th positions of miRNA, respectively. Furthermore, when considering only sites within the top 33% of miRNA targeting scores (miRanda >130) (Enright et al. 2003), a more pronounced peak of CIMs can also be found at position corresponding to the beginning of miRNAs (Supplemental Fig. S3B).

As described above, there is a ∼2 nt shift between the peak position of crosslinking-induced deletions and substitutions in piRNA and miRNA CLASH data. To further examine whether this trend can be observed from the identical sets of small RNA targets, we limited our analysis to those hybrids where both substitutions and deletions were found at the same small RNA targeting sites. We found that position of deletion preferentially occurs mostly at 2 nt upstream of the position of substitution on mRNAs (Supplemental Table S2A).

Together, our analyses suggest that the CIMs are preferentially produced on piRNA or miRNA targets at regions corresponding to the center of the small RNAs, and in some cases also at the beginning of the small RNAs. The similarity in CIM distribution between miRNA and piRNA Argonautes reinforces the idea that CIMs serve as molecular footprints of Argonaute interactions with their target mRNAs (Feng et al. 2019).

### CIMs are also enriched at nucleotides within piRNA target sites that contain local mismatches

**FIGURE 3.**
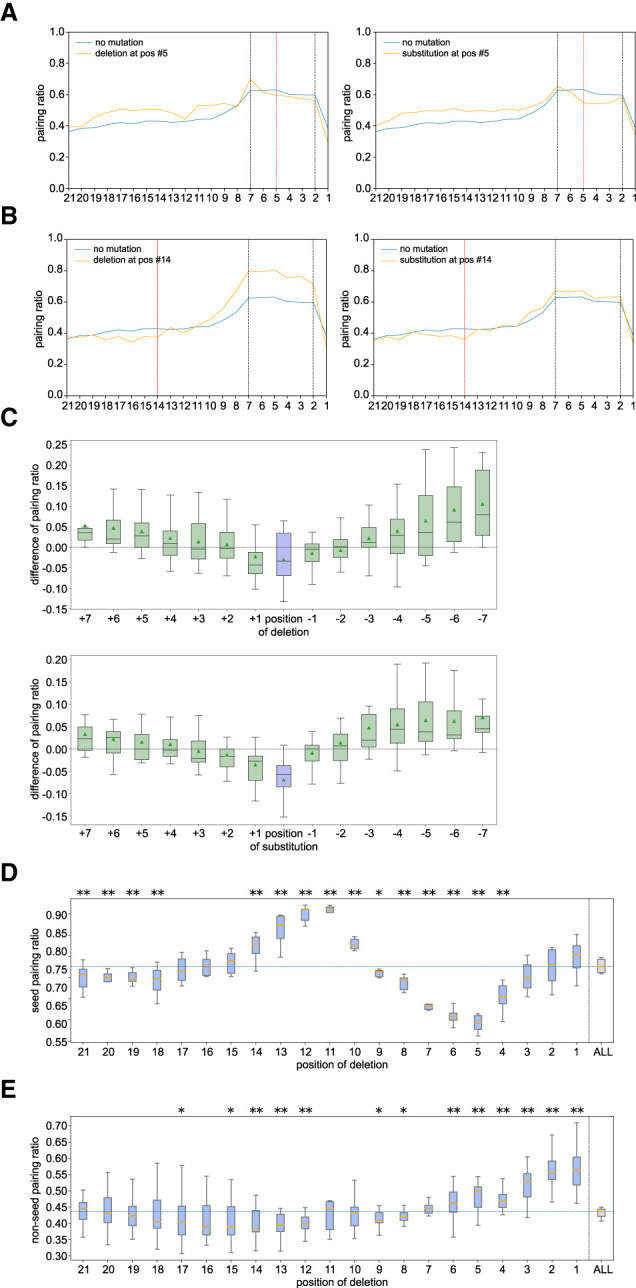
CIMs preferentially occur at positions with local piRNA pairing mismatches. (*A*) Pairing ratios between mRNA and piRNA at the indicated position (pos) from hybrids containing mRNA deletions (*left*) or substitutions (*right*) at position 5 (red line) compared to hybrids without mutations (orange lines) in PRG-1 PIWI CLASH data. (*B*) Pairing ratio of mRNA and piRNAs at the indicated position (pos) of piRNAs from hybrids containing mRNA deletion (*left*) or substitution (*right*) at position 14 (red line) compared to hybrids without mutations (orange lines) in PRG-1 PIWI CLASH data. (*C*) Differences in pairing ratios around CIMs in PRG-1 PIWI CLASH data. The differences were calculated by subtracting the pairing ratios of hybrids with deletions (*top*) or substitutions (*bottom*) from the pairing ratios of all hybrids. The analysis was centered on the CIM location (position 0) and extended to the indicated upstream and downstream nucleotides. Lines display median values, boxes display first and third quartiles, and whiskers display fifth and 95th percentiles, respectively. (*D*) Differences in pairing ratio at the seed region between hybrids with deletion at the indicated position and hybrids with CIMs in PRG-1 PIWI CLASH data. Lines display median values, boxes display first and third quartiles, and whiskers display fifth and 95th percentiles, respectively. (*) *P* < 0.05 and (**) *P* < 0.01. (*E*) Differences in pairing ratio at the non-seed region between hybrids with deletion at the indicated position and hybrids with CIMs in PRG-1 PIWI CLASH data. Lines display median values, boxes display first and third quartiles, and whiskers display fifth and 95th percentiles, respectively. (*) *P* < 0.05 and (**) *P* < 0.01.

Base-pairing at the seed region, which is the second to seventh or eighth nucleotides of piRNA or miRNA, is known to play a critical role in piRNA and miRNA targeting in *C. elegans* (Broughton et al. 2016; Zhang et al. 2018). As we noticed that CIMs are somewhat depleted in their seed regions (Fig. 2A,B), we wondered whether the local base-pairing between small RNAs with mRNAs could affect the distribution of CIMs. To examine the relationship between CIM position and base-pairing, we combined all hybrids with CIMs corresponding to each piRNA position from PIWI CLASH data and calculated the base-pairing ratio for each position and then compared those ratios from hybrids without CIMs. We noticed a trend that the piRNA base-pairing ratio is reduced around the location of the CIMs. For example, hybrids with mRNA deletion or substitution at the position corresponding to the fifth nucleotide of the piRNA exhibits a reduced pairing ratio around the fifth nucleotide when compared to all hybrids (Fig. 3A). Similarly, hybrids with CIMs at the 14th nucleotide exhibit a reduced pairing ratio around 14th position (Fig. 3B). This global trend of reduced piRNA base-pairing ratios near the location of CIMs was further demonstrated when we aligned all CIMs at the center (position 0) and compared piRNA to the pairing ratio around the CIMs. Indeed, we found that the piRNA base-pairing ratios were reduced around the location of CIMs (Fig. 3C). However, in ALG-1 miRNA CLASH data, such trends could only be slightly found in the substitution, but not the deletion, carrying hybrids (Supplemental Fig. S4A). Our analyses reveal that CIMs preferentially occur at nucleotides of piRNA targeting sites with less stable local base-pairing.

### CIM analysis reveals that base-pairing in the non-seed regions complements for seed mismatch in piRNA target recognition

In addition, we noticed a relationship between CIM locations and the overall piRNA pairing ratio at seed and non-seed regions: When CIMs occur at non-seed regions, such as position 14, we observed an overall increased pairing ratio at nucleotides within the seed region, accompanied by slightly decreased or no-change pairing ratios at nucleotide of the non-seed region (Fig. 3B). On the contrary, when CIMs occur at the seed region, such as position 5, these target sites exhibit an increased pairing ratio at nucleotides in the non-seed region, but a decreased or no change in pairing ratios at nucleotides within the seed region (Fig. 3A). We then systematically compared the location of CIM position and overall piRNA pairing ratio at the seed region (position 2–7) or non-seed region (8–21). We found that hybrids with CIMs at some of the non-seed positions, especially those with mRNA deletion at the position corresponding to the 11th and 12th of piRNAs or mRNA substitution at the position corresponding to the first, ninth, and 10th of piRNA, exhibit the highest pairing ratios at the seed region when compared to all hybrids carrying CIMs (Fig. 3D; Supplemental Fig. S4B). On the contrary, we noticed that hybrids with CIMs at several positions within the seed positions exhibit a reduced pairing ratio at the seed region but also an increased pairing ratio at the non-seed region (Fig. 3D,E; Supplemental Fig. S2B,C). These observations suggest that hybrids with CIMs at the center or beginning regions are associated with target sites that exhibit higher seed pairing ratios. In contrast, hybrids with CIMs in the seed region are associated with lower seed pairing ratios, potentially representing noncanonical piRNA target sites. Notably, in these noncanonical sites with CIMs, the reduction in seed pairing was accompanied by an increase in base-pairing at the non-seed regions (Fig. 3E; Supplemental Fig. S4C), likely compensating to facilitate stable piRNA binding.

For ALG-1 miRNA CLASH data in *C. elegans*, we also observed a decreased pairing ratio at the seed regions for those hybrids with CIMs located at several positions within the seed region position, such as positions between fourth and seventh (Supplemental Fig. S4D,E), but we did not observe a clear increase of non-seed pairing for those hybrids (Supplemental Fig. S4D,E). Somewhat similar to piRNAs, hybrids with deletions and substitutions in the middle region exhibited a slightly increased base-pairing ratio at the seed region (Supplemental Fig. S4D).

### Target sites with CIMs exhibit stronger regulatory effects

**FIGURE 4.**
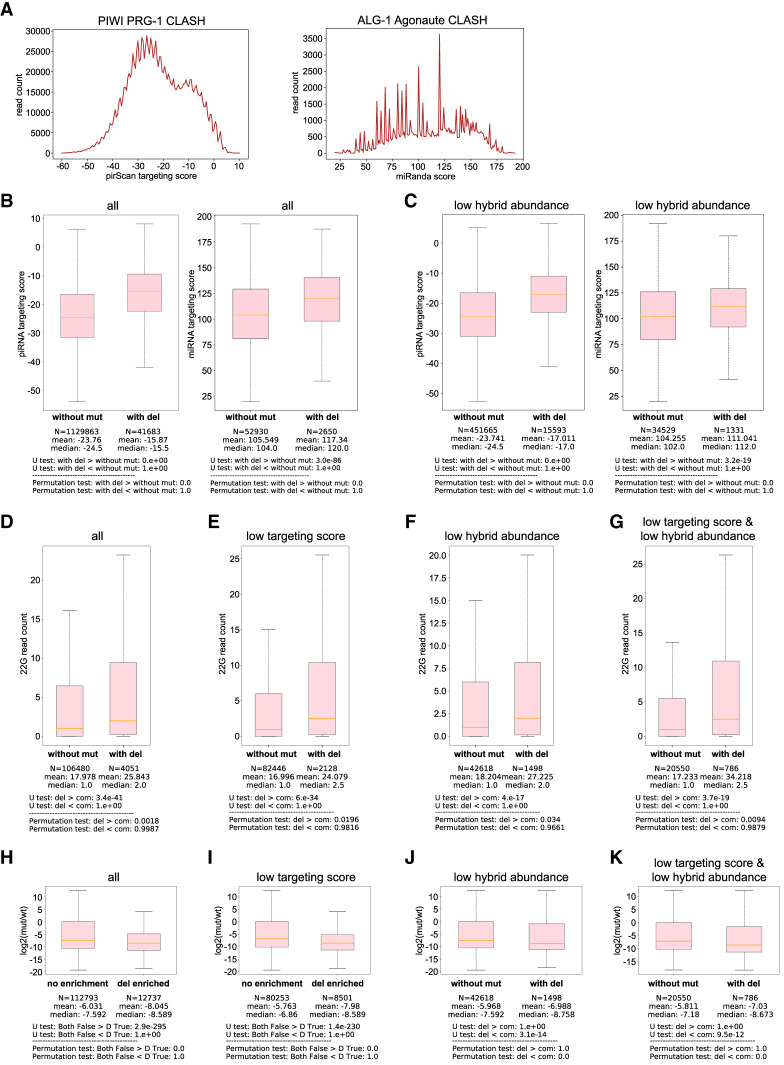
piRNA targets with CIMs exhibit stronger regulatory effects. (*A*) Distribution of piRNA targeting score (pirScan) and miRNA targeting score (miRanda) from the hybrids of PRG-1 piRNA CLASH data (*left*) and ALG-1 miRNA iCLIP data (*right*), respectively. (*B*) Comparison of piRNA targeting scores (*left*) or miRNA targeting scores (*right*) between those target sites without mutation and those with deletion. The statistical significance (*P*-value) of the difference in fold change was calculated by the Mann–Whitney *U* test and the permutation test. (*C*) Comparison of piRNA targeting scores (*left*) or miRNA targeting scores (*right*) of target sites with low abundant reads between those without mutation and those with deletion. The statistical significance (*P*-value) of the difference in fold change was calculated by the Mann–Whitney *U* test and the permutation test. (*D*) Comparison of local WAGO-1 22G-RNAs levels of piRNA targeting sites between those without mutation and those with deletion. The 22G-RNA levels mapped within the 100 nt window centered at the piRNA targeting site were calculated. The statistical significance (*P*-value) of the difference in fold change was calculated by the Mann–Whitney *U* test and the permutation test. (*E*–*G*) Comparison of local WAGO-1 22G-RNAs levels of piRNA targeting sites with poor targeting scores (sites with pirScan < −15) (*E*), low abundant reads (*F*), or both (*G*) between those without mutation and those with deletion. The 22G-RNA levels that mapped within the 100 nt window centered at the piRNA targeting site were calculated. The statistical significance (*P*-value) of the difference in fold change was calculated by the Mann–Whitney *U* test and the permutation test. (*H*) The ratio of local WAGO-1 22G-RNAs levels of piRNA targeting sites in the *prg-1* mutant over those in wild type of all piRNA targeting sites between those without mutation and those with deletion. The statistical significance (*P*-value) of the difference in fold change was calculated by the Mann–Whitney *U* test and the permutation test. (*I*–*K*) The ratio of local WAGO-1 22G-RNAs levels of piRNA targeting sites in the *prg-1* mutant over those in wild type of piRNA targeting sites with poor targeting scores (miRanda score < 100) (*I*), low abundant reads (*J*), or both (*G*) between those without CIMs and those with deletion. The statistical significance (*P*-value) of the difference in fold change was calculated by the Mann–Whitney *U* test and the permutation test.

Currently, the abundance and pairing score/pairing energy of hybrids from CLASH data are the two main characteristics used to identify candidates of functionally relevant small RNA targeting sites (Helwak et al. 2013; Shen et al. 2018; Ariura et al. 2024). Previous analyses have reported that the hybrids identified from piRNA and miRNA CLASH data exhibit various base-pairing modes (Broughton et al. 2016; Shen et al. 2018; Ariura et al. 2024); however, the available piRNA or miRNA targeting score algorithms mainly reward canonical seed perfect base-pairing (Enright et al. 2003; Wu et al. 2018). When we analyzed the *C. elegans* piRNA and miRNA targets identified through CLASH data (Fig. 4A), we found that most hybrids do not contain canonical piRNA or miRNA regulatory sites (pirScan score > 0, or miRanda score > 140). In addition, over 39% of piRNA target sites and 60% of miRNA target sites are represented by only a single hybrid (Supplemental Table S4A,B). Thus, although binding energy and hybrid read abundance are critical criteria for identifying functionally relevant targeting sites, these metrics alone are likely insufficient, as most binding sites lack canonical binding sites and/or are supported by very few reads.

Since we observed that functional noncanonical target sites for the miRNA *let-7* and the piRNA *21ur-1563* contain CIMs (Fig. 1C), we hypothesized that small RNA target sites identified by CLASH that contain CIMs are more likely to be functionally relevant. As expected, we observed that small RNA targeting sites supported by low-, medium-, or high-abundance hybrids from PIWI piRNA CLASH and ALG-1 CLIP show a positive correlation with their respective piRNA or miRNA pairing scores (Supplemental Fig. S5A,B). Similarly, we found hybrids carrying CIMs exhibit higher piRNA and miRNA pairing scores than those without CIMs (Fig. 4B; Supplemental Fig. S5C). Notably, when we limit our analysis to those target sites of low hybrid abundance (sites represented by only a single hybrid), CIM-containing hybrids also displayed higher pairing scores than those without CIMs (Fig. 4C; Supplemental Fig. S5D). These observations support that CIM-containing sites are enriched for functional interactions and may complement existing characteristics, including hybrid abundance, to evaluate small RNA targeting sites.

Encouraged by these observations, we directly compared the transcriptome-wide regulatory effects of hybrids with or without CIMs. In *C. elegans*, piRNAs induce gene silencing of their target through the production of secondary small RNAs, known as WAGO 22G-RNAs, locally at piRNA-targeted sites (Bagijn et al. 2012; Lee et al. 2012). We therefore used the local WAGO-1 Argonaute-associated 22G-RNA levels at piRNA target sites as a proxy to evaluate the functional relevance of piRNA targeting sites. In this analysis, we examined the level of WAGO 22G-RNAs mapped to germline-silenced mRNAs (WAGO targets), which are known to be regulated by piRNAs (Zhang et al. 2018; Cornes et al. 2022; Wu et al. 2023). As expected, WAGO-22G-RNA abundance was higher at small RNA targeting sites with stronger pairing scores or at sites represented by more hybrid reads (Supplemental Fig. S5E,F), showing that both pairing score and hybrid abundance positively correlate with local 22G-RNA production.

We found that there are significantly more WAGO-1 22G-RNAs produced around piRNA targeting sites from those hybrids with CIMs than from those without (Fig. 4D). Notably, this increase of WAGO-22G-RNAs from CIMs carrying sites persists even when the analysis is restricted to sites with low piRNA targeting scores (pirScan < −15), low hybrid abundance (hybrid read count = 1), or both poor targeting scores and low hybrid abundance (Fig. 4E–G; Supplemental Fig. S5G–J). Although these changes are statistically significant for hybrids containing crosslinking-induced deletions, hybrids containing cross-linking-induced substitutions at sites with low hybrid abundance exhibit a similar trend but reach significance only by *t*-test, not by the more stringent permutation test. These observations showed that piRNA targeting sites with CIMs in general elicit more downstream silencing signals.

We then evaluated how much WAGO-1-associated 22G-RNA production at the piRNA targeting sites is contributed by piRNA targeting. If a target site is strongly regulated by piRNAs, we expect a greater reduction of 22G-RNA level in the *prg-1* mutant, which loses all piRNAs. As expected, a great reduction of 22G-RNA level is found at small RNA targeting sites with stronger pairing scores and at sites represented by more hybrid reads (Supplemental Fig. S5K,L), showing that both pairing score and hybrid abundance positively correlate with local 22G-RNA production. Similarly, we found a significantly greater reduction of WAGO-1 22G-RNA level for those targeting sites from hybrids with CIMs than those without (Fig. 4H; Supplemental Fig. S5M). Similar observations were found for those hybrids with poor targeting scores, with low hybrid abundance, and with both poor targeting score and low hybrid abundance (Fig. 4I–K; Supplemental Fig. S5N–P). These observations further show that piRNA targeting sites within hybrids containing CIMs are generally subject to stronger regulation by the piRNA pathway compared to those without CIMs, including those cases where read abundance and/or targeting score would otherwise provide limited confidence.

**FIGURE 5.**
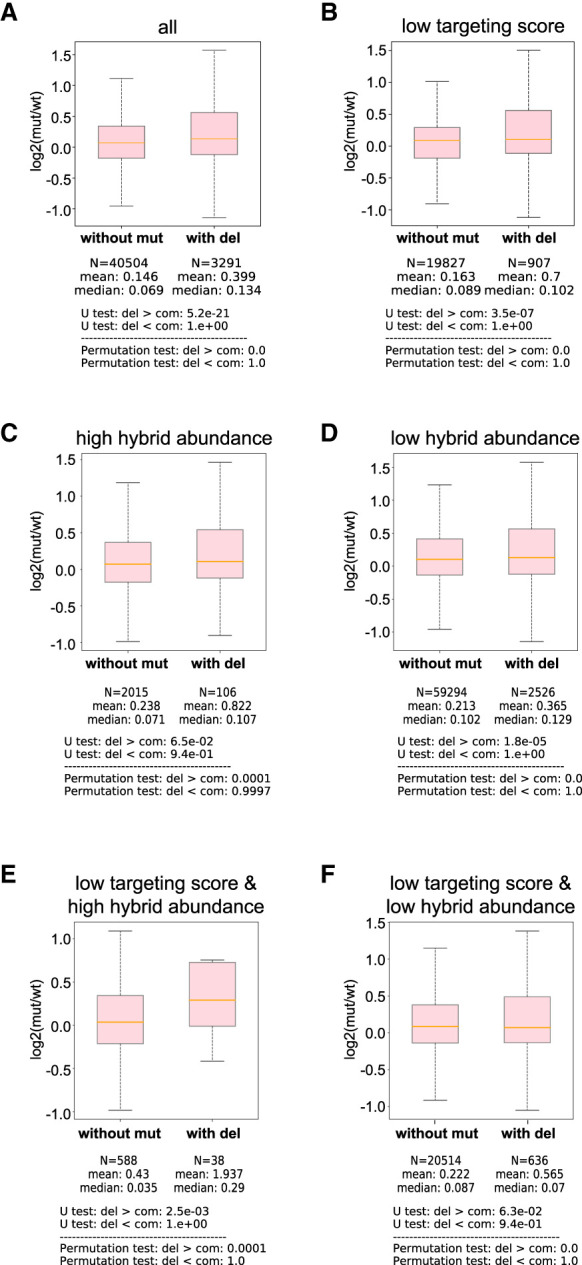
miRNA targets with CIMs exhibit stronger regulatory effects. (*A*) The ratio of mRNA levels in the *alg-1* mutant over those in wild type of all miRNA targeting sites between those without CIMs and those with deletion. The statistical significance (*P*-value) of the difference in fold change was calculated by the Mann–Whitney *U* test and the permutation test. (*B*) The ratio of mRNA levels in the *alg-1* mutant over those in wild type of miRNA targeting sites with poor targeting scores (miRanda score <100) between those without CIMs and those with deletion. The statistical significance (*P*-value) of the difference in fold change was calculated by the Mann–Whitney *U* test and the permutation test. (*C*–*F*) The ratio of mRNA levels in the *alg-1* mutant over those in wild type of miRNA targeting sites with high abundance reads (*C*), low abundance reads (*D*), both poor targeting scores and high abundance reads (*E*), and both poor targeting scores and low abundance reads (*F*) between those without CIMs and those with deletion. The statistical significance (*P*-value) of the difference in fold change was calculated by the Mann–Whitney *U* test and the permutation test.

**FIGURE 6.**
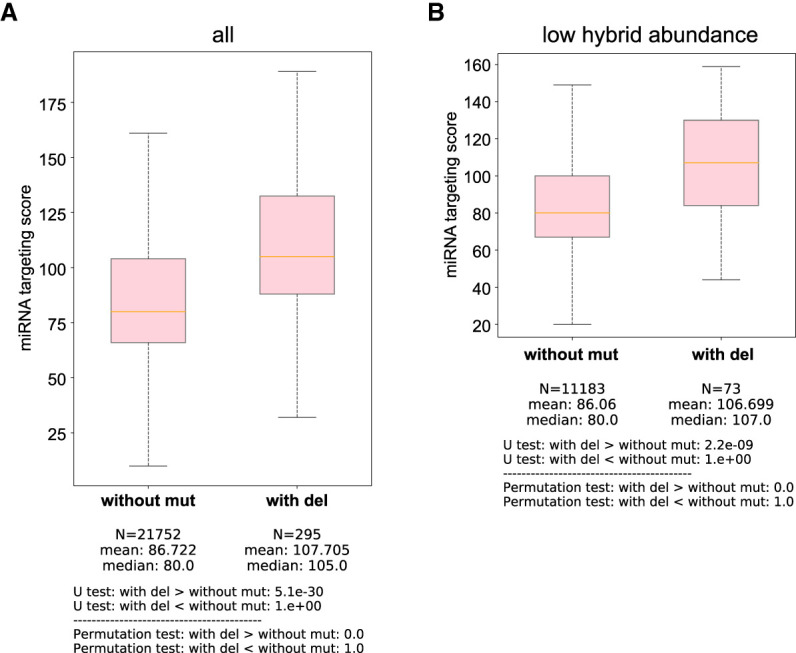
CIMs containing miRNA targeting sites identified from human AGO-1 CLASH data sets overall display better miRNA targeting scores than those sites without CIMs. (*A*) Comparison of miRNA targeting scores between those without mutation and those with deletion from the hybrids of human AGO-1 miRNA CLASH data. The statistical significance (*P*-value) of the difference in fold change was calculated by the Mann–Whitney *U* test and the permutation test. (*B*) Comparison of miRNA targeting scores with low abundant reads between those without mutation and those with deletion from the hybrids of human AGO-1 miRNA CLASH data. The statistical significance (*P*-value) of the difference in fold change was calculated by the Mann–Whitney *U* test and the permutation test.

We then compared miRNA targeting sites from hybrids with CIMs to those without CIMs for their ability to regulate mRNA expression. Since miRNAs can trigger the mRNA degradation of their targets, we use mRNA levels as a proxy for evaluating the effects of miRNA-mediated gene regulation (Brown et al. 2017). In miRNA target sites without CIMs, we observed a global increase in target mRNA expression in the *alg-1* mutant, as expected. miRNA target sites identified through hybrids containing CIMs, whether deletions or substitutions, exhibited a significantly greater increase in mRNA levels in the *alg-1* mutant compared to those without CIMs (Fig. 5A; Supplemental Fig. S6A). This trend was also observed for miRNA sites with poor miRNA targeting scores (miRanda score <140) (Fig. 5B; Supplemental Fig. S6B). Notably, the impact of CIMs was more pronounced for target sites supported by higher read counts (greater than five reads) than for those represented by low read counts (single read) (Figs. 5C–F, 6C–F). Given that miRNAs primarily fine-tune rather than silence gene expression, these results suggest that while CIMs mark genuine crosslinking sites, a threshold level of interaction frequency—reflected by hybrid read abundance—may be required to elicit detectable changes in mRNA levels.

Together, our analyses support the notion that CIMs containing hybrids are enriched for functional piRNA and miRNA target sites, even those hybrids with weak canonical targeting features and/or low hybrid abundance. Our analysis highlights that CIMs provide additional hybrid characteristics, in addition to hybrids abundance and targeting score/energy, to access the functional significance of small RNA sites in CLASH data.

### CIMs are present in human AGO-1 miRNA clear-sequencing data sets

To assess whether MUTACLASH can also enhance the analysis of CLASH data generated from another organism, we examined human AGO-1 miRNA CLEAR (a CLASH-like method) (Moore et al. 2015). We found that CIMs, including deletions and substitutions, are also detectable in human AGO-1 data sets (Supplemental Table S3A). While only deletions but not substitutions display a strong preference for uridine-derived mutations (Supplemental Table S3B), hybrids containing CIMs—including those with low hybrid abundance (single-read hybrid)—showed significantly better pairing scores than those without CIMs in the human AGO1 CLEAR-CLIP data set (Fig. 6A,B; Supplemental Fig. S7A,B). To facilitate researchers in searching miRNA targets in vivo, we have recently built a database that collects the miRNA target sites from various miRNA CLASH data from worm, mice, and human, where the location and read counts of CIMs are reported (Yang et al. 2025).

### Crosslinking-induced deletions in non-hybrid reads provide additional insights into small RNA targeting sites

As described earlier, both CLASH and CLASH-like experiments generate not only hybrid reads but also non-hybrid (mRNA-only) reads containing CIMs (Supplemental Tables S1, S3). Although non-hybrid reads do not directly identify the specific small RNA responsible for targeting, regions of mRNAs enriched for CIMs are likely to represent authentic crosslinking sites regulated by the corresponding small RNA class and thus highlight functionally relevant targets.

**FIGURE 7.**
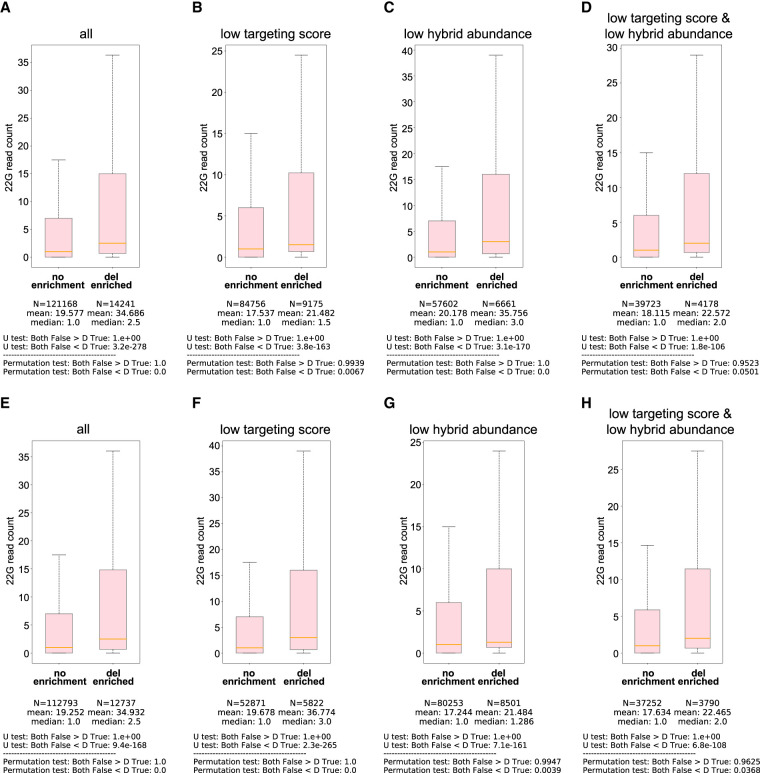
mRNA regions enriched for crosslinking-induced deletions in non-hybrid reads of CLASH data exhibited stronger regulatory effects than those without CIM enrichment. (*A*) Comparison of WAGO-1 22G-RNAs from target sites of the PRG-1 PIWI CLASH data between mRNA regions not enriched for deletion to those enriched for deletion. The statistical significance (*P*-value) of the difference in fold change was calculated by the Mann–Whitney *U* test and the permutation test. (*B*–*D*) Comparison of WAGO-1 22G-RNAs from target sites of the PRG-1 PIWI CLASH data with poor targeting scores (sites with pirScan < −15) (*B*), low abundant reads (*C*), or both (*D*) between mRNA regions not enriched for deletion to those enriched for deletion. The statistical significance (*P*-value) of the difference in fold change was calculated by the Mann–Whitney *U* test and the permutation test. (*E*) Comparison of target sites not carrying CIMs for the WAGO-1 22G-RNAs from the PRG-1 PIWI CLASH data between mRNA regions not enriched for deletion to those enriched for deletion. The statistical significance (*P*-value) of the difference in fold change was calculated by the Mann–Whitney *U* test and the permutation test. (*F*–*H*) Comparison of target sites not carrying CIMs of WAGO-1 22G-RNAs from the PRG-1 PIWI CLASH data with poor targeting scores (sites with pirScan < −15) (*F*), low abundant reads (*G*), or both (*H*) between mRNA regions not enriched for deletion to those enriched for deletion. The statistical significance (*P*-value) of the difference in fold change was calculated by the Mann–Whitney *U* test and the permutation test.

To explore this possibility, we first mapped CIM locations from non-hybrid reads and determined whether the mRNA target regions of individual hybrids were enriched for CIMs. Notably, while hybrids with mRNA regions enriched for crosslinking-induced deletions in non-hybrid reads exhibited stronger regulatory effects than those without CIM enrichment, this trend was not consistently observed for substitutions (Fig. 7A; Supplemental Fig. S8A). This pattern is consistent with previous analyses of CLIP data, which showed that deletions, but not substitutions, more faithfully mark crosslinking sites of RNA-binding proteins (Zhang and Darnell 2011), likely due to the higher background rate of substitutions in mRNA sequencing.

For piRNA, we observed stronger regulatory effects from hybrids of different groups, including hybrids of low targeting scores and/or low hybrid abundance if the mRNA regions are enriched for crosslinking-induced deletion (Fig. 7B–D). For miRNA, similar to CIM analysis of hybrids, we observed stronger regulatory effects from hybrids of different groups, including hybrids of low targeting scores, low hybrid abundance, or low targeting score but with high hybrid abundance if the mRNA regions are enriched for crosslinking-induced deletion (Fig. 7B–F).

We next asked whether hybrids lacking CIMs themselves, but whose mRNA target regions are enriched for crosslinking-induced deletions in non-hybrid reads, also show enhanced regulatory effects. Indeed, our analyses confirmed this trend for both *C. elegans* piRNA and miRNA hybrids (Fig. 7E–H; Supplemental Fig. S8G–K). Because crosslinking-induced deletions occur more frequently in non-hybrid reads than in hybrids, these CIM analyses from non-hybrid reads provide additional candidates for functionally relevant sites.

We have now integrated this feature into the MUTACLASH pipeline, providing an additional annotation column indicating whether the mRNA target region of each hybrid is enriched for crosslinking-induced deletions in non-hybrid reads. It should be noted that since mRNA regions enriched for crosslinking-induced deletions reveal only the sites of protein–mRNA crosslinking, without identifying the corresponding regulatory small RNA, such information should be considered supplementary evidence rather than direct validation of specific small RNA–mRNA interactions. Nonetheless, these findings suggest that analyzing the distribution of crosslinking-induced deletions from non-hybrid reads offers complementary and valuable insights for interpreting CLASH data sets.

## DISCUSSION

In this manuscript, we developed the MUTACLASH pipeline to identify and analyze CIMs in CLASH data. We examined the location, distribution, and functional implication of CIMs in the miRNA and piRNA CLASH data in *C. elegans* (Broughton et al. 2016; Shen et al. 2018). Our data revealed that CIMs are present in these libraries and that the CIM pattern on mRNAs represents miRNA Argonaute and PIWI footprints on their target mRNAs. We also reported an ∼2 nt shift between crosslinking-induced deletions and substitutions, which likely reflects the dynamics of reverse transcriptase (RT) enzyme processing the modified RNA template. One possible model is that deletions occur when the RT enzyme stalls completely at the crosslinked site, leading to the omission of a few nucleotides. In contrast, substitutions arise slightly downstream, as the RT may incorporate aberrant nucleotides 1–2 bases after bypassing the crosslink.

Given that CIMs serve as molecular footprints of RNPs, our CIM analysis provides critical molecular and mechanistic insights into PIWI and Argonaute target recognition. Specifically, the enrichment of CIMs at the central region of PIWI and Argonaute binding sites likely reflects structural constraints, such as a relatively narrow binding channel that facilitates base-pairing between the small RNA and mRNA. Alternatively, this enrichment may result from the close proximity of catalytic residues within PIWI/Argonaute to the target mRNA nucleotides corresponding to the 10th and 11th positions of the small RNA (Sheu-Gruttadauria and MacRae 2017; Anzelon et al. 2021; Li et al. 2025). The enrichment of CIMs at nucleotides with local mismatches between mRNA and piRNA base-pairing likely reflects how RNA bulges created by mismatches expose surrounding nucleotides to direct interactions with the PIWI/Argonaute protein. Critically, this observation supports the plasticity of PIWI PRG-1 protein to adopt alternative conformations, bringing mismatched nucleotides from different pairing regions into closer proximity to its protein surface. Notably, although piRNA targeting sites with CIMs in their seed region exhibit a decreased pairing ratio within the seed, we observed an increased pairing ratio in the non-seed regions. This suggests that enhanced non-seed pairing can compensate for imperfect seed base-pairing in piRNA target recognition (Fig. 5). This finding aligns with recent reports in several animals indicating that base-pairing across the entire piRNA sequence contributes to PIWI target recognition, supporting a more flexible and distributed mode of interaction (Priyadarshini et al. 2022; Gainetdinov et al. 2023; Ariura et al. 2024; Li et al. 2024).

Together, our CIM analysis reveals PIWI's structural flexibility, which enables it to tolerate mismatches in target recognition. This flexibility is likely crucial for PIWI's role in genome defense. Since transposons rapidly accumulate mutations, a rigid sequence-recognition mechanism would restrict PIWI's ability to silence evolving transposon-derived transcripts. Instead, by adopting alternative conformations that accommodate mismatches, PIWI maintains specificity while ensuring robust recognition of sequence variants. This adaptive mechanism allows PIWI to effectively silence transposons despite their genetic variability, thereby safeguarding genome integrity.

Currently, hybrid abundance and pairing score or energy derived from CLASH data are the primary characteristics used to identify functionally relevant small RNA–target interactions. However, these features alone are imperfect indicators, since only a small fraction of CLASH data corresponds to canonical small RNA targeting sites, and most targeting sites are represented by only a few hybrid reads. Our analyses demonstrate that CLASH hybrids containing CIMs exhibit significantly stronger regulatory effects than those without CIMs, including targets with poor targeting scores and/or low hybrid abundance. Additionally, while non-hybrid mRNA reads from CLASH data have typically been overlooked in previous analyses, our CIM analysis suggests that crosslinking-induced deletion in non-hybrid mRNAs with CIMs represents Argonaute-crosslinked mRNAs in vivo. Therefore, CIM analysis of both hybrid and non-hybrid mRNAs in CLASH data provides valuable insights into mRNA targets that are regulated by small RNAs in vivo, expanding our ability to identify functional interactions beyond canonical base-pairing rules.

Accurate annotation of CIMs in PIWI CLASH data requires careful consideration of transposon sequence diversity. Because DNA transposons often exist as multiple, highly similar copies with sequence polymorphisms, aligning hybrids or non-hybrid reads to an incomplete or single-consensus transposon reference can lead to false identification of crosslinking-induced mutations. Including all available sequences of DNA transposon families in the genome helps minimize such misannotation and ensures that CIMs reflect genuine crosslinking events rather than inherent sequence variance among transposon copies.

While CIMs provide valuable information for identifying Argonaute–RNA interaction sites, they are present in only a small fraction of total hybrid reads and display a nucleotide preference for uridines at their origin. Consequently, CIMs are not detected at all genuine small RNA–target interactions, and their absence should not be interpreted as evidence of nonfunctionality. Nonetheless, both CIMs within hybrids and crosslinking-induced deletions—which occur more frequently—serve as informative markers of Argonaute crosslinking events. Our analyses demonstrate that incorporating CIM analysis can complement existing analytical approaches for assessing the functional relevance of small RNA targeting sites, revealing candidate target sites that might otherwise be ignored by traditional CLASH.

Taken together, MUTACLASH integrates CIM analysis with traditional metrics such as pairing strength and hybrid abundance, providing a comprehensive framework for identifying biologically meaningful small RNA target sites.

## MATERIALS AND METHODS

### MUTACLASH pipeline

MUTACLASH pipeline can be used to process raw reads from CLASH experiments to identify in vivo small RNA binding sites. In brief, the adapter and barcode sequences from the raw reads from worm PRG-1 or human AGO-1 data sets were trimmed using cutadapt (version: 2.10) with the following command: -m 17 -M 70 -q 30 -a. Raw reads from the worm ALG-1 data set were trimmed using trim_galore (version 0.6.5) with the following command: ‐‐length 17 ‐‐dont_gzip -q 30 ‐‐max_length 70. Reads were deduplicated, and their read counts were calculated using the custom Python scripts. The hybrid reads consisting of piRNA and mRNA transcripts were identified by ChiRA algorithm (Videm et al. 2021) using BWA-MEM aligner with the following commands: -b -p 4 -l1 12 -go1 6 -mm1 4 -s1 18. The *C. elegans* mRNA transcripts (WormBase WS275 version) and piRNA sequences from WormBase (WS275 version) and type 2 piRNA sequence (Gu et al. 2012) were used as reference sequences. Once the hybrids are identified, the mutated positions on mRNAs were obtained through reading the MD tag with a custom Python script and SAMtools (Danecek et al. 2021). Mutations identified in overlapping regions of mRNA and small RNA sequences are removed to avoid misinterpretation, as these mutations can be derived from either small RNA or mRNA. The precise location of the mutations on mRNAs is reported. When aligning to transposon sequences, we recommend including the -a parameter in hybrid identification and mapping process to ensure that all alignment results are reported. This option allows comprehensive evaluation of potential target sites while minimizing loss of relevant hybrid information during the mapping process. After mapping of hybrid reads, a filtering step is implemented using a custom Python script to check whether a reported CIM has an alternative, perfectly aligned mapping; such cases are excluded to avoid misannotation of CIMs. A custom Python script is then used to calculate the nucleotide composition and percentage of each mutation type of CIMs.

### Definition of mRNA target sites and aggregation of hybrid reads

To define mRNA targeting sites, hybrids sharing the same small RNA and identical predicted mRNA target region were considered to represent a common target site. For each mRNA targeting site, the read counts of all hybrids are aggregated, as well as the read counts of hybrids containing CIM, deletion or substitution, were identified and aggregated to evaluate the relationship between CIM presence and hybrid abundance. This approach enabled quantitative comparison of CIM-containing hybrids and total hybrid reads across defined mRNA target sites.

Hybrids were categorized into high, medium, and low groups based on their pairing scores and hybrid abundance. Unless specifically specified, the high-, medium-, and low-pairing groups were defined as the top, middle, and bottom one-third of hybrids ranked by pairing score, respectively. Hybrid abundance was classified as high for normalized read counts greater than or equal to five, medium for two to four reads, and low for a single read.

### Map the location of CIMs within the predicted small RNA targeting sites

When the mRNA interacting sequences (CLASH-identified regions) are shorter than miRNA or piRNA, they are first extended to the size of miRNA and piRNAs using both the upstream and downstream sequences before they are examined for sites with the best pairing energy/score. The predicted miRNA or piRNA binding sites are defined with miRanda (John et al. 2004) or pirScan (Wu et al. 2018), respectively. The following commands are used for distinct tools: pirScan commands: ‐‐ex n^[d]^, miRanda commands: -sc 0. Custom Python scripts are used for plotting and analysis, including converting the absolute location of mutation into relative location in the small RNA targeting sites, plotting the read count distribution, visualizing the mutation ratio at each position, and generating graphs for the pairing ratio and abundance results.

### Statistical assessment of CIM enrichment in small RNA target from non-hybrid reads

To assess whether CIMs were enriched within the mRNA target region of each hybrid, we compared the read counts of CIMs (either deletions or substitutions) within the identified target region to those across the entire mRNA sequence. Statistical significance was evaluated using the Mann–Whitney *U* test implemented in scipy.stats.mannwhitneyu() in Python with default settings, where enrichment is considered significant at *P* ≤ 0.01.

### Measurements of WAGO-1 22G-RNAs levels at piRNA targeting sites

The *C. elegans* transcriptome data (WS275) annotation was used for mapping *C. elegans* reads. WAGO targets (*n* = 3644) are defined as transcripts whose mapped 22G-RNAs exhibit over twofold enrichment of 22G-RNAs from WAGO-1 IP than that from input sample (Gu et al. 2009).

For measurements of 22G-RNA reads around piRNA targeting sites, the 50 nt (±25 nt) window centered at the 10th nucleotide of piRNA sequence was used to calculate the read count number of the 22G-RNA reads mapped to these regions.

### mRNA level analysis from RNA-seq

FASTQ reads were trimmed of adaptors using cutadapt (Martin 2011). Trimmed reads were aligned to the *C. elegans* genome build WS275 using bowtie2 version 2.3.0 (Langmead and Salzberg 2012). After alignment, reads were overlapped with genomic features (protein-coding genes, pseudogenes, transposons) using bedtools intersect (Quinlan and Hall 2010). Reads per kilobase million (RPKM) values were then calculated for each individual feature by summing the total reads mapping to that feature, multiplied by 1 × 10^6^ and divided by the product of the kilobase length of the feature and the total number of reads mapping to protein-coding genes.

### Data sets

The iCLIP data of ALG-1 (SRR3882949), the CLASH data of PRG-1 (SRR6512652) (Broughton et al. 2016; Shen et al. 2018), and the clear-seq of human AGO-1 (combination of libraries from SRR2413156-59) (Moore et al. 2015) were used in these analyses. WAGO-1-associated small RNA data (SRR8482951/WT, SRR8482949/*prg-1* mutant) were used for analysis of WAGO-1 22G-RNA levels around piRNA targeting sites (Barucci et al. 2020). mRNA-seq data of wild-type (SRX2826535, SRX2826536, SRX2826587) or from the *alg-1*(gk214) mutant (SRX2826541, SRX2626542, and SRX2826543) were used (Brown et al. 2017).

## DATA DEPOSITION

The MUTACLASH pipeline and all custom scripts used in this study are available at GitHub deposit link: https://github.com/lu1215/MutaCLASH. Additional scripts for CIM analysis can be found at https://github.com/lu1215/Regenerate-MutaCLASH-paper-figure. The comprehensive data sets of hybrid reads and CIM locations for *C. elegans* piRNA and miRNA interactions are provided at http://nas.csblab.ee.ncku.edu.tw:32200/sharing/iXKXJG1yb. All sequencing data analyzed in this study are accessible through the NCBI GEO or ENA databases, with specific SRR numbers provided in the Materials and Methods and Data sets section.

## SUPPLEMENTAL MATERIAL

Supplemental material is available for this article.

## Figures and Tables

**Wei-Sheng Wu:**
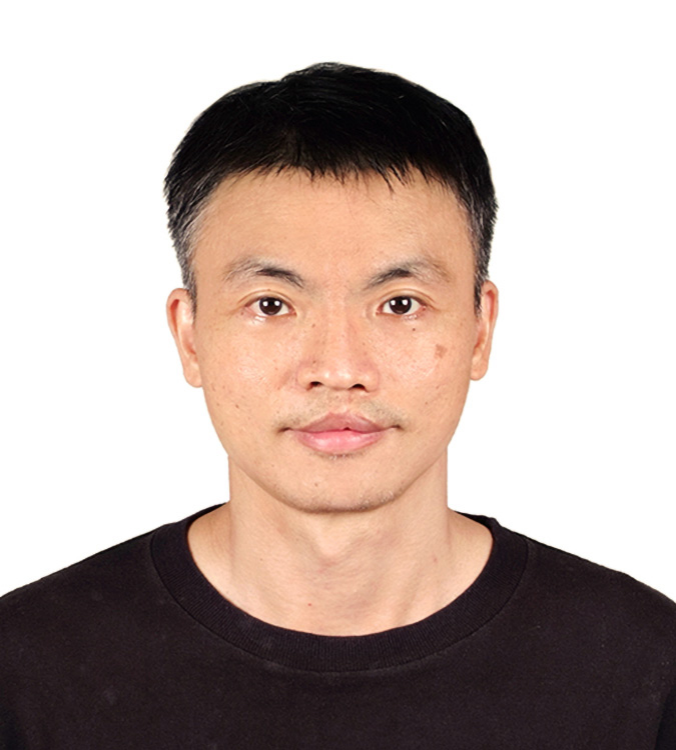


## MEET THE FIRST AUTHOR

***Meet the First Author(s)* is an editorial feature within *RNA*, in which the first author(s) of research-based papers in each issue have the opportunity to introduce themselves and their work to readers of *RNA* and the RNA research community. Wei-Sheng Wu is the first author of this paper, “MUTACLASH: identifying functional small RNA target sites using crosslinking-induced mutations.” Professor Wu is a computational systems biologist. His specialty is to develop computational tools/databases to extract biological insights from biological raw data.**

**What are the major results described in your paper and how do they impact this branch of the field?**

This study introduces MUTACLASH, a bioinformatics tool designed to analyze crosslinking-induced mutations (CIMs) in CLASH data sets to better understand small RNA–mRNA interactions. Using data from *C. elegans* miRNA and piRNA CLASH experiments, we found that CIMs serve as precise molecular footprints marking Argonaute binding sites on target mRNAs. These CIMs are enriched at the centers of small RNA-binding regions and within mismatched target nucleotides in piRNA interactions. Moreover, mRNAs showing noncanonical binding sites or low hybrid abundance but marked by CIMs display stronger regulatory effects. The findings demonstrate that CIM profiling can reveal functional small RNA target sites that traditional computational approaches might miss.

**What led you to study RNA or this aspect of RNA science?**

With advancements in next-generation sequencing (NGS), bioinformaticians play important roles in mining the information embedded in the raw reads. In collaboration with Dr. Heng-Chi Lee (the corresponding author of this paper), I started to analyze the NGS data of small noncoding RNAs in *C. elegans*. I am looking forward to solving more biological problems related to RNAs using computational approaches.

**During the course of these experiments, were there any surprising results or particular difficulties that altered your thinking and subsequent focus?**

In this study, we hypothesized that CIMs can serve as reliable molecular footprints of Argonaute and PIWI binding, allowing functional small RNA–target interactions to be distinguished from noise. To test this hypothesis, we made a lot of effort in developing MUTACLASH, a bioinformatics tool designed to analyze CIMs in CLASH data sets. To our surprise, we found that CIMs are predominantly localized at the centers of small RNA binding regions and at mismatched nucleotides within piRNA target sites. Furthermore, mRNAs with noncanonical binding sites or low hybrid abundance but that carry CIM marks exhibit stronger regulatory outcomes. We also make MUTACLASH an easy-to-use software tool to allow RNA researchers to easily analyze CIMs in their CLASH data sets in the future.

**What are some of the landmark moments that provoked your interest in science or your development as a scientist?**

I was originally trained as an electrical engineer. During my PhD studies, I changed my research field from control theory to computational systems biology. My career goal is to use control theory to not only understand how a cell operates but also to gain the ability to control a cell's behavior. Since RNA biology has emerged as one of the most influential fields in biological/biomedical research, I will continue studying the biological functions of all kinds of small noncoding RNAs using computational approaches.

**What are your subsequent near- or long-term career plans?**

My career goal is to continue creating valuable computational tools that address biological challenges. Through collaborative projects with researchers from various biological fields, I aim to explore and resolve significant unanswered questions in biology. Contributing meaningfully to the scientific community gives me a deep sense of fulfillment.
